# Analysis of health care and actual needs of patients with psoriasis: a survey on the Italian population

**DOI:** 10.1186/1471-2458-7-59

**Published:** 2007-04-21

**Authors:** Emma Altobelli, Mara Maccarone, Reimondo Petrocelli, Ciro Marziliano, Alberto Giannetti, Ketty Peris, Sergio Chimenti

**Affiliations:** 1 Department of Internal Medicine and Public Health, University of L'Aquila, Coppito-Delta 6, 67100 L'Aquila, Italy; 2 Italian Association of Psoriatic Patients (ADIPSO), Rome, Italy; 3 Department of Dermatology, University of Modena and Reggio Emilia, Italy; 4 Department of Dermatology, University of L'Aquila, Italy; 5 Department of Dermatology, University of Rome Tor Vergata, Italy

## Abstract

**Background:**

Over recent years the public health system has shown increasing interest in patients' views for use as guideline criteria in evaluating the quality of assistance above all for those patients with chronic diseases. Hence the interest in psoriasis, which is a chronic disease frequently associated with diabetes mellitus, hypertension, obesity, and cardiovascular diseases. The aims of our study were to describe clinic characteristics of patients with psoriasis, the quality of the assistance perceived by patients arrived at outpatients clinics and the information received, in order to identify areas in Italy requiring improvement.

**Methods:**

1954 patients, aged between 18 and 85 years, were consecutively enrolled at outpatients clinics across 21 Italian provinces over the period December 2004 – January 2006. A standardized questionnaire was developed in collaboration with an Italian Association of Psoriatic Patients (A.DI.PSO) and tested in a pilot study. The questionnaire was divided into three sections: the first section included social, demographic and individual variables; the second concerned the quality of the assistance perceived by the patients at public dermatologic clinics and the third focused on the need of information requirements of patients with psoriasis. The χ^2 ^test was used to estimate the association between the categorical variables under study. Kruskal-Wallis test was applied to the interval and ordinal variables.

**Results:**

The presence of psoriatic arthritis was reported in 26.0% of patients. Associated chronic diseases included depression (15.4%), hypertension (13.3%), obesity (8.9%) and type 2 diabetes mellitus (7.3%). The study highlighted the need of improvements of health care services at public dermatologic clinics especially in overcoming architectonic barriers and reducing appointment wait-times, particularly in South Italy. However, patients reported a positive relationship with Health System employers due to the confidentiality. This positive impression was confirmed by the observation that dermatologists were considered the best source of information about therapies on psoriasis.

**Conclusion:**

Our study allowed to identify critical aspects which could be tackled through initiatives with the aim of improving these emerged needs.

## Background

Psoriasis is a chronic disease with a different prevalence between countries varying from 0.8 to 3.1% [1,2]; psoriatic arthritis (PsA) has been found to be associated with skin lesions in 20–34% of patients [3-5]. Psoriasis has a significant involvement on patients' quality of life and their social and family relationships [6,7]. In addition, psoriasis has proven to be frequently associated with chronic extra-cutaneous diseases such as diabetes mellitus (DM), hypertension, obesity, and cardiovascular diseases [8-10].

Over recent years, the public health system has shown increasing interest in the quality of medical care as well as in the patients' degree of satisfaction which in turn represents a useful indicator of the quality of health care. In fact, it is known that a positive perception of medical services by the patients, a good relationship between patients and medical staff and a comfort of the surroundings improve the doctor-patient relationship leading to enhanced therapeutic compliance, better quality of the health service and saving of economical resources. Furthermore, the level of information given to the patient about their disease may improve disease management and hence quality of life.

The aims of our study were to describe: i) the clinic characteristics of patients with psoriasis, ii) the quality of medical care as perceived by the patients at public dermatologic clinics, and iii) the information received by the patient, in order to identify areas in Italy with priority need of improvement.

## Methods

### Study population

The study population consisted of 1954 patients, aged 18–85 years, who were consecutively enrolled into the study over the period 1^st ^December 2004 – 31^st ^January 2006: 991 patients were males with an average age of 48.4 years (standard deviation [sd] 15.0) and 963 were females with an average age of 47 years (standard deviation [sd] 15.0). Patients attended the public dermatologic clinics taking part in the project across 21 Italian provinces: Ascoli Piceno, Bari, Benevento, Bologna, Brindisi, Brescia, Catania, Catanzaro, Cesena-Forlì, Firenze, L'Aquila, Lucca, Milano, Modena, Napoli, Palermo, Padova, Prato, Reggio-Calabria, Roma, Verona. To improve the efficiency of the sampling plan, stratified sampling by province was used: to estimate the sampling dimension the following parameters were used: sample error E = 0.025, the event occurrence proportion p = 0.5 (in the case of maximum variability), probability 1-α = 0.95.

### Data collection

A standardized questionnaire, developed in close collaboration with an Italian Association of Psoriatic Patients (Associazione per la Difesa dei Pazienti Psoriasici, A.DI.PSO) and tested in a pilot study, was used for data collection. The questionnaire was explained by trained personnel to the patients when they underwent routine visits at dermatologic clinics; patients completed the questionnaire singularly and autonomously. The questionnaire was divided into three parts: the first section concerned patients' social, demographic and case history variables such as smoking and alcohol consumption, associated diseases such as depression, hypertension, type 2 DM and obesity. All diagnoses were reported by patients: they were asked to provide the age of psoriasis (defined as the age of patients at the first dermatologic visit for psoriasis) while diagnoses of associated disease was conceivably established by other specialists but not verified by us. The second part focussed on the quality of the assistance perceived by the patients at public dermatologic clinics and took into account parameters such as the accessibility of the dermatologic clinic (e.g. the presence of architectonic barriers), the programming of routine visits, the time spent in the waiting room of the clinic, the quality of the waiting room (e.g. comfort, privacy etc.), and the third part looked at the information needs of the patients.

Informed consent was obtained from all subjects. All of the subjects who were invited to participate in the study agreed to do so.

### Statistical analysis

The data collection was analyzed by grouping the patients in the participating provinces into three geographical areas: North, Centre and South.

The χ^2 ^test was used to estimate the association between the categorial variables under study. Kruskal-Wallis test was applied to the interval and ordinal variables. A value of p < 0.05 was considered statistically significant. SAS software was used for the statistical analyses [11].

## Results

The results of the first section of the questionnaire are summarized in tables 1,2,3. No differences were found for marital status between the three areas: North, Centre and South Italy. However a statistically significant difference was detected concerning education (p < 0.0001). Out of 1947 patients interviewed, 2.7% had not received education. The lower level of education was observed in the South whilst the highest level of education was found in the Centre (p = 0.0006). A statistically significant difference was also found for occupational status; the highest level of unemployment was detected in the South (p < 0.0001). Our results show that the Centre Italy has the highest number of working days lost compared to the North and South Italy (p = 0.002).

**Table 1:** Distribution of social and demographic variables in the three Italian areas (North, Centre and South)

	North		Centre		South		p
	No.	%	No.	%	No.	%	
**Marital status (total no. cases 1954)**							
Never married	203	28.5	194	31.5	162	25.9	0.09
Married	510	71.5	422	68.5	463	74.1	
**Education (total no. cases 1947)**							
No education	17	2.4	7	1.1	28	4.5	0.0006
Primary school	105	14.7	73	11.7	124	19.8	
Junior high school	175	24.6	154	25.1	140	22.4	
High school	301	42.2	286	46.5	244	39.1	
University	115	16.1	96	15.6	82	14.2	
**Occupational status (total no. cases 1954)**							
Manual worker	128	18.0	97	15.8	97	15.5	< 0.0001
Office worker	150	21.0	149	24.2	132	21.1	
Professional	165	23.1	122	19.8	114	18.2	
Unemployed	17	2.4	32	5.2	68	11.0	
Houseworker	62	8.7	57	9.2	54	8.6	
Retired	118	16.6	102	16.6	77	12.3	
Other	73	10.2	57	9.2	83	13.3	
**Working days lost (total no. cases 1235)**							
1–7	205	46.6	136	34.6	176	43.8	0.002
8–14	82	18.6	85	21.6	95	23.6	
15–21	35	8.0	42	10.7	41	10.2	
22–28	16	3.6	19	4.8	16	4.0	
29–35	32	7.3	59	15.1	29	7.2	
> 35	70	15.9	52	13.2	45	11.2	

**Table 2:** Distribution of smoking and alcohol consumption in the 3 Italian areas

Age-group (years)	20–39				40–59				> = 60			
Italy	North No. %	Centre No. %	South No. %	p	North No. %	Centre No. %	Suth No. %	p	North No. %	Centre No. %	South No. %	p

**Cigarettes per day (total no. of cases: 759)**												
< 5	14 14.1	5 4.6	16 23.5	0.0005	6 6.1	18 15.4	12 9.2	0.11	6 11.8	3 13.6	9 15.0	0.90
5–15	61 61.6	58 53.7	24 35.3		43 43.9	42 35.9	55 42.0		21 41.2	11 50.0	23 38.3	
16–20	10 10.1	23 21.3	11 16.2		31 31.6	33 28.2	28 21.4		10 19.6	4 18.2	9 15.0	
> 20	14 14.2	22 20.4	17 25.0		18 31.4	24 20.5	36 27.4		14 27.4	4 18.2	19 31.7	
**Wine consumption (total no. of cases :857)**												
1–2 glasses/day	56 82.3	56 80.0	36 83.7	0.87	99 72.3	104 68.9	98 76.0	0.42	70 66.0	57 83.8	69 81.2	0.001
> 2 glasses/day	12 17.7	14 20.0	7 16.3		38 27.7	47 31.1	31 24.0		36 34.0	11 16.2	16 18.8	
**Beer consumption (total no. of cases: 233)**												
1–2 glasses/day	36 87.8	33 91.7	16 84.2	-	28 90.3	20 83.3	34 82.9	-	11 91.7	5 100.0	20 83.3	-
> 2 glasses/day	5 12.2	3 8.3	3 15.8		3 9.7	4 16.7	7 17.1		1 8.3	0 0.0	4 16.7	
**Spirits (total no. of cases: 178)**												
1 drink/day	12 70.6	13 81.2	4 30.8	0.01	16 72.7	17 51.5	23 67.6	0.21	17 85.0	4 80.0	9 50.0	-
> 1 drink/day	5 29.4	3 18.8	9 69.2		6 27.3	16 48.5	11 32.4		3 15.0	1 20.0	9 50.0	

P values were calculated for the items whose expected values were more than 5 for 80% of cells and none expected value less than 1.

**Table 3:** Distribution of extra-cutaneous disease associated with psoriasis in the 3 Italian areas

	**Males (total no. of cases: 991)**				**Females (total no. of cases: 963)**			
Disease category (ICD^§ ^code number)	North No. (%)	Centre No. (%)	South No. (%)	p	North No. (%)	Centre No. (%)	South No. (%)	p

Depression (296)								
Present	34 (8.6)	56 (18.7)	43 (14.6)		59 (18.7)	53 (16.8)	57 (17.2)	
Absent	363 (91.4)	244 (81.3)	251 (85.4)	0.0004	257 (81.3)	263 (83.2)	274 (82.8)	0.81
Myalgia (729.1)								
Present	0 (0.0)	2 (0.7)	3 (1.0)		0 (0.0)	6 (1.9)	7 (2.1)	
Absent	397 (100.0)	298 (99.3)	291 (99.0)	-	316 (100.0)	310 (98.1)	324 (97.9)	-
Obesità (278)								
Present	39 (9.8)	23 (7.7)	27 (9.2)		29 (9.2)	32 (10.1)	34 (10.3)	
Absent	358 (90.2)	277(92.3)	267 (90.8)	0.61	287 (98.8)	284 (89.9)	297 (89.7)	0.88
Type 2 DM (250)								
Present	29 (7.3)	15 (5.0)	21 (7.19)		23 (7.3)	12 (3.8)	43 (13.0)	
Absent	368 (92.7)	285 (95.09)	273 (92.9)	0.42	293 (92.7)	304 (96.2)	288 (87.0)	< 0.0001
Hypertension (401)								
Present	71 (17.9)	52 (17.3)	38 (12.9)		41 (13.0)	24 (7.6)	35 (10.6)	
Absent	326 (82.19	248 (82.7)	256 (87.1)	0.18	275 (87.0)	292 (92.4)	296 (89.4)	0.08
Heart disease(410–414)								
Present	7 (1.8)	2 (0.7)	14 (4.8)		1 (0.3)	0 (0.0)	7 (2.1)	
Absent	390 (98.2)	298 (99.3)	280 (95.2)	0.003	315 (99.7)	316 (100.0)	324 (97.9)	-
Herpes virus (054)								
Present	5 (1.3)	11 (3.7)	10 (3.4)		18 (5.7)	15 (4.7)	10 (3.0)	
Absent	392 (98.7)	289 (96.3)	284 (96.6)	0.08	298 (94.3)	301 (95.3)	321 (97.0)	0.25
Other *								
Present	2 (0.5)	4 (1.3)	10 (3.4)		6 (1.9)	9 (2.8)	6 (1.8)	
Absent	395 (99.5)	296 (98.7)	284 (96.6)	0.01	310 (98.1)	307 (97.2)	325 (98.2)	0.61

^§ ^ICD = IX International Classification Diseases

* iritis (364), lupus (710.0)

P values were calculated for the diseases whose expected values were more than 5 for 80% of cells and none expected value less than 1.

Overall, 45.4% of the patients were smokers; 45.2% drunk wine, 11.9% beer and 9.1% spirits. The distribution of smokers and drinkers was analyzed for the three geographical areas (table 2). The heaviest smokers (> 20 cigarettes per day) were in the South in the 20–39 age group (25.0%, p = 0.0006). There was no significant difference between the three areas in the group of patients 40–59 years of age. The heaviest wine consumption (> 2 glasses/day) in those that drink was in the North in the ≥ 60 age group (p = 0.001). Beer (> 2 glasses/day) and spirit (< 2 glass/day) consumption was greater in South Italy in the 20–39 age group being 15.8% and 69.2%, respectively.

PsA was reported in 26.0% of patients. The most frequent extra-cutaneous diseases associated with psoriasis were depression (15.4%), hypertension (13.3%), obesity (8.9%) and type 2 DM (7.3%). PsA was associated with obesity in 36% of cases (p = 0.0007), type 2 DM in 34% of cases (p = 0.03), hypertension in 32% of cases (p = 0.02) and depression in 30% of cases although the latter result was not statistically significant. Table 3 shows the distribution of the extra-cutaneous diseases associated with psoriasis by stratified area and gender. The distribution of depression differed between North, Centre and South Italy in men with the highest percentage being 18.7% (p = 0.0004) in Centre Italy. No significant distribution was observed for hypertension between North, Centre or South or in men or women. Type 2 DM in female patients was higher in the South (13.0%, p = 0.02).

The results of the second part of the questionnaire concerning the quality of medical services perceived by the patients at public dermatologic clinics are summarized in table 4. Better access in terms of lack of architectonic barriers was registered in the North as compared to the South (p < 0.0001). The number of routine visits was higher in the North (every 30 days) than in the South (once a year) (p < 0.0001). However patients in the North changed dermatologic clinics more frequently than patients in the South (p < 0.0001). Patients attending dermatologic clinics in the North were more satisfied by levels on accessibility, time (minutes) spent in the waiting room and quality of the time in the waiting room (p < 0.0001). In addition, patients attending dermatologic clinics in the North were more satisfied by levels of confidentiality and privacy and by the levels of helpfulness and courtesy of the health system personnel with respect to patients attending clinics in the South (p < 0.0001). The results concerning the patients' knowledge about psoriasis and need of information are summarized in table 5. Patients in the North were more satisfied with the explanation of their disease by their dermatologist and general practitioner (p < 0.0001). There was also a statistically significant difference between the three geographical areas as far as the information source on disease treatment was concerned. Patients living in the Centre felt they needed more information concerning therapy (p < 0.0001). Notably, only 29.5% of patients were aware of patients' rights and there was no significant difference between North, Centre and South Italy. Finally, the knowledge of homeopathy and herbal products was more widespread in the South (p < 0.0001) as was the knowledge of therapies such as acupuncture and the use of phototherapy (p = 0.005).

**Table 4:** Distribution of quality of the assistance perceived by patients in the 3 Italian areas

Item	Total responders		North		Centre		South		p
	No	%	No	%	No	%	No	%	
Clinic accessibility									
Bad	1813	92.8	50	7.5	48	8.5	134	22.9	< 0.0001
Poor			66	9.9	109	19.4	115	19.7	
Good			289	43.4	229	40.7	236	40.4	
Very good			261	39.2	117	31.4	99	17.0	
Routine visits every									
15 days	1792	91.7	36	5.5	25	4.4	36	6.3	< 0.0001
30 days			197	30.1	84	14.9	100	17.5	
2–4 months			187	28.6	199	35.2	146	25.4	
5–7 months			117	17.9	140	24.8	125	21.8	
1 year			117	17.9	117	20.7	166	29.0	
Length of time at the same clinic									
< 6 months	1792	91.7	158	23.4	111	19.8	110	19.8	0.01
12 months			136	20.1	98	17.5	75	13.4	
18 months			112	16.6	78	13.9	84	15.1	
24 months			69	10.2	100	17.9	116	20.8	
30 months			49	7.3	42	7.5	36	6.5	
> 30 months			151	22.4	131	23.4	136	24.4	
Time (minutes) spent in the waiting room									
< 15'	1856	95.0	194	28.5	113	19.6	138	23.0	< 0.0001
15–30'			316	46.5	249	43.2	184	30.7	
35–60'			139	20.4	114	19.8	153	25.5	
> 60			31	4.6	100	17.4	125	20.8	
Quality of the time spent in the waiting room									
Unacceptable	1834	93.9	28	4.2	68	12.0	119	20.1	< 0.0001
Poor			84	12.4	120	21.2	128	21.7	
Good			439	64.8	271	47.9	290	49.1	
Very good			126	18.6	107	18.9	54	9.1	
Confidentiality and privacy of the clinic personnel									
Poor	1873	95.9	34	4.9	101	17.2	149	25.0	< 0.0001
Good			293	42.5	236	40.1	259	43.5	
Very good			362	52.6	251	42.7	188	31.5	
Helpfulness and courtesy of the clinic personnel									
Excellent	1868	95.4	326	47.0	261	44.8	177	29.9	< 0.0001
Good			306	44.1	194	33.3	196	33.2	
Sufficient			49	7.1	103	17.7	126	21.3	
Poor			13	1.8	25	4.2	92	15.6	
Overall level of services offered by the public health system									
Unsatisfactory	1831	93.7	36	5.2	105	18.4	165	28.8	< 0.0001
Satisfactory			334	48.6	259	45.4	267	46.5	
Very good			317	46.2	306	36.2	142	24.7	

**Table 5:** Patients' knowledge about psoriasis and information need distributed in the 3 Italian areas

Item	Total responders		North		Centre		South		p
	No	%	No	%	No	%	No	%	
Patient's opinion of doctor's explanation of the health problem									
Positive	1875	96.0	528	76.3	364	62.0	330	55.4	< 0.0001
Negative			59	8.5	97	16.5	114	19.1	
Don't know			105	15.2	126	21.5	152	25.5	
Patient's view of the best information source on disease treatment									
Dermatologist	1870	95.7	434	63.2	333	56.7	292	49.0	< 0.0001
General practitioner			162	23.6	60	10.2	137	23.0	
Patient association			30	4.3	132	22.5	66	11.1	
Other			61	8.9	62	10.6	101	16.9	
Patient's view as to whether patients require more information concerning therapy									
Yes	1884	96.4	607	88.1	575	96.5	482	80.5	< 0.0001
No			82	11.9	21	3.5	117	19.5	
Patient's opinion of the best information source on psoriasis									
General practitioner	1842	94.3	148	23.0	113	19.0	121	20.0	< 0.0001
Pharmacist			21	3.3	6	1.0	44	7.3	
Dermatologist			265	41.2	207	34.8	197	32.6	
Illustrated medication leaflet			7	1.1	7	1.2	19	3.2	
Health personnel			49	7.6	42	7.1	39	6.5	
Friends and family			13	2.0	5	0.8	16	2.6	
Health magazines			30	4.7	17	2.9	33	5.5	
Books			6	0.9	1	0.2	6	1.0	
Internet			25	3.9	53	8.9	42	7.0	
Newspapers			7	1.1	11	1.8	11	1.8	
Information campaigns			42	6.5	34	5.7	20	3.3	
Patient associations			19	3.0	95	16.0	34	5.6	
Other			11	1.7	4	0.6	22	3.6	
Knowledge of patients' rights									
Yes	1726	88.3	154	26.0	178	30.8	178	32.0	0.06
No			438	74.0	400	69.2	378	68.0	
Knowledge of homeopathic medication and herb-based products									
Yes	1855	94.9	124	19.1	123	20.6	187	30.7	< 0.0001
No			526	80.9	473	79.4	422	69.3	
Knowledge of therapies such as acupuncture, the use of phototherapy									
Yes	1848	94.6	159	24.5	150	25.4	195	32.0	0.005
No			489	75.5	441	74.6	414	68.0	

## Discussion

This study is the first one in Italy to be carried out using a questionnaire developed in close collaboration with a National Psoriasis Patient Association named A.DI.PSO. This methodological choice was based on the assumption that suggestions from the Association would correspond more closely to the actual needs of psoriatic patients. In fact, as expected, the data from this study allowed us to identify areas in Italy requiring improvement. It emerged that preventive programmes are required for risk factors such as smoking and drinking [12-15], especially in central and southern Italy for smokers in the 20 – 59 age groups and for drinkers (> 2 glasses/day) in the North in the ≥ 60 age group.

The frequency of PsA in our study population was 26% which is within the range reported by other authors [3-5,16]. The highest frequency of PsA was in the North (48.1%) and in men. The presence of PsA complicates disease management due to both its physical and emotional impact: PsA often makes simple every day activities difficult whilst on an emotional level can cause anxiety and depression [17]. The complexity of management of psoriasis can also be aggravated, as in our population series, by the concomitant occurrence of other diseases such as obesity in 36% of patients, type 2 DM (34%) and hypertension (32%) and depression (30%). The distribution of obesity and hypertension did not differ for geographical area or gender, whilst type 2 DM had a higher frequency in females in the South. The association of such diseases with psoriasis is often due to nutritional factors such as a high-calorie diet. In fact, improvement of psoriasis in places with an insufficient food supply (e.g. prison camps) has been reported [18,19]. If the problem therefore lies with lifestyle, then the approach to be adopted by the public health system should focus on health education and health promotion as means of prevention.

A further aspect we investigated in this study was the quality of the assistance perceived by the patient at public dermatologic clinics on an organizational and comfort level. Although it was difficult to establish minimum standards owing to a lack of recent studies on a national level, a demand nevertheless emerged for improvements in patient reception in public services. This demand focussed on overcoming architectonic barriers and reducing time the patient spent in the waiting room especially in South Italy. It also emerged that patients in the North were more satisfied with the relationship with health staff as shown by the patient's positive impression about the confidentiality, privacy, helpfulness and courtesy of the health staff. This positive aspect was also confirmed by the fact that the general practitioner and the dermatologist in particular were considered the best source of information about their disease by the patients. Previous studies showed that a good doctor-patient relationship is the most important factor in determining patient's satisfaction [20-22]. Continuous improvements in the doctor-patient relationship is an important aim as it leads to better therapeutic compliance; in fact doctors can learn to change their style of communication as other studies have previously shown [23,24].

Interestingly, patients relied significantly on no-profit A.DI.PSO association for inquiry about their disease. Such information were indeed considered more helpful than those provided by campaigns promoted by the public health system.

## Conclusion

In conclusions, the results of our study showed that there is a good basis, such as the patient-doctor relationship for initiatives aimed at improving the outcome of some of the indicators used in this study.

## Competing interests

The author(s) declare that they have no competing interest.

## Authors' contributions

EA, the principal investigator, designed the study, performed statistical analyses, interpreted the data, and wrote the article. MM participated in the development of the questionnaire and the data collection. RP participated to statistical analysis and literature search. CM created data-base and archived data. KP participated in the development of the questionnaire and clinical interpretation of data and critically revised the manuscript. AG participated in the development of the questionnaire and data collection. SC participated to test the questionnaire in the pilot study and data collection.

All authors read and approved the final manuscript.

## Pre-publication history

The pre-publication history for this paper can be accessed here:


